# Molecular characterization of hepatitis C virus in liver disease patients in Botswana: a retrospective cross-sectional study

**DOI:** 10.1186/s12879-019-4514-1

**Published:** 2019-10-22

**Authors:** Lynnette Bhebhe, Motswedi Anderson, Sajini Souda, Wonderful T. Choga, Edward Zumbika, Zachary M. Shaver, Tshepiso Mbangiwa, Bonolo B. Phinius, Chabeni C. Banda, Pinkie Melamu, Rosemary M. Musonda, Max Essex, Jason T. Blackard, Sikhulile Moyo, Simani Gaseitsiwe

**Affiliations:** 1 Botswana Harvard AIDS Institute Partnership, Research Laboratory, Gaborone, Botswana; 2 Department of Applied Biology and Biochemistry, National University of Science and Technology, Bulawayo, Zimbabwe; 3 Department of Pathology, Faculty of Medicine, University of Botswana, Gaborone, Botswana; 4 Department of Medical Laboratory Sciences, Faculty of Health Sciences, University of Botswana, Gaborone, Botswana; 5 Department of Immunology and Infectious Diseases, Harvard T.H. Chan School of Public Health, Boston, MA USA; 6 University of Cincinnati College of Medicine, Cincinnati, OH USA

**Keywords:** Hepatitis C virus, HCV, Genotypes, Mutations, DAA, Botswana

## Abstract

**Background:**

Hepatitis C virus (HCV) infection is a major cause of chronic liver disease globally. Direct acting antivirals (DAAs) have proven effective in curing HCV. However, the current standard of care (SOC) in Botswana remains PEGylated interferon-α (IFN-α) with ribavirin. Several mutations have been reported to confer resistance to interferon-based treatments. Therefore, there is a need to determine HCV genotypes in Botswana, as these data will guide new treatment guidelines and understanding of HCV epidemiology in Botswana.

**Methods:**

This was a retrospective cross-sectional pilot study utilizing plasma obtained from 55 participants from Princess Marina Hospital in Gaborone, Botswana. The partial core region of HCV was amplified, and genotypes were determined using phylogenetic analysis.

**Results:**

Four genotype 5a and two genotype 4v sequences were identified. Two significant mutations – K10Q and R70Q – were observed in genotype 5a sequences and have been associated with increased risk of hepatocellular carcinoma (HCC), while R70Q confers resistance to interferon-based treatments.

**Conclusion:**

Genotypes 5a and 4v are circulating in Botswana. The presence of mutations in genotype 5 suggests that some patients may not respond to IFN-based regimens. The information obtained in this study, in addition to the World health organization (WHO) recommendations, can be utilized by policy makers to implement DAAs as the new SOC for HCV treatment in Botswana.

## Background

Viral hepatitis is the seventh leading cause of death globally [1], and hepatitis C virus (HCV) is one of the leading causes of liver failure. The prevalence of HCV differs by geographical location. In Africa, a range in prevalence from 1.7 to 14.7% has been reported [2–5]. In Botswana, preliminary studies reported a low HCV seroprevalence of below 1% [6–8] although Botswana is endemic for both human immunodeficiency virus (HIV) and hepatitis B virus (HBV) with the prevalence of HIV/HBV co-infection ranging from 4 to 10.6% [6, 7].

Treatment of HCV has evolved rapidly with the introduction of direct acting antivirals (DAA) in the last decade. DAAs can achieve sustained virologic responses (SVR) of 95 to 100% [9], have shortened treatment duration, and have overcome many challenges faced by administering interferon-based regimens. The World health organization (WHO) has recommended that DAA regimens be used for HCV treatment, and the choice of antiviral therapy and duration of treatment of the DAAs are dependent on HCV genotype. Therefore, studies of circulating HCV genotypes are important prior to widescale treatment rollout [10]. In Botswana, there are no data on HCV genotypes. This study aimed to determine the HCV genotypes circulating in Botswana and to identify clinically relevant mutations within the HCV core region.

## Methods

This is a retrospective cross-sectional pilot study utilizing 55 stored plasma samples collected between February 2015 and July 2016 from 55 liver disease patients at the liver clinic of Princess Marina Hospital, a referral hospital in Gaborone, Botswana. The study was conducted at Botswana Harvard AIDS Institute Partnership.

HCV ribonucleic acid (RNA) was extracted using the Qiagen Viral RNA kit using 140 μl plasma samples according to the manufacturer’s specifications (Qiagen, Hilden, Germany). The extracts were stored at − 80 °C prior to genotyping. Amplification targeted the partial core region with two primer sets – outer core primers (5′ – ACT GCC TGA TAG GGT GCT TGC – 3′, nt 288 → 308 and 5′ – ATG TAC CCC ATG AGG TCG GC – 3′, nt 732 ← 751) and inner core primers (5′ – AGG TCT CGT AGA CCG TGC A – 3′, nt 321 → 339 and 5′ – CAT GTG AGG GTA TCG ATG AC – 3′, nt 705 ← 724) previously described, [11, 12] relative to the H77 reference [13]. Nested PCR was carried out using Superscript (III) one-step RT-PCR with Platinum Taq DNA polymerase high fidelity kit (Invitrogen, USA) according to the manufacturer’s instructions. PCR products were visualized on 1.5% agarose gel stained with ethidium bromide, and the positive amplicons were purified using the ZR DNA sequencing clean up kit (Zymo, Irvine, CA, USA) according to the manufacturer’s specifications. Population sequencing was conducted using BigDye Terminator version 3.0 kit (Applied Biosystems; Foster City, CA, USA) sequencing chemistry on a 3130XL ABI genetic analyser (ABI PRISM 3130XL; Applied Biosystems).

Sequence files were edited using Sequencher version 5.0 software (Gene Codes Corp., Ann Arbor, MI, USA). Phylogenetic analysis was used to evaluate HCV genotypes and included reference sequences from the Los Alamos HCV sequence database (https://hcv.lanl.gov/content/index). Sequences were aligned in Clustal X version 2.1. Additional phylogenetic inference was conducted using the Bayesian Markov chain Monte Carlo (MCMC) in the Bayesian Evolutionary Sampling Trees (BEAST) version 1.7.5 as described previously [8, 14]. Posterior probabilities > 80% were deemed statistically significant. Sequences were exported to BioEdit software where nucleotide sequences were translated to amino acids. H77 (accession number AF009606 [13]) served as the reference sequence, and mutations were visually analysed per amino acid position. Importantly, H77 is a genotype 1a reference; therefore, several genotype 1 references together with genotype 4 and 5 reference sequences were included in the analysis to distinguish between polymorphic regions that differ by genotype and true mutations. Sequences were submitted to National Center for Biotechnology Information (NCBI) GenBank under accession numbers MK392625 to MK392630. Statistical analysis was performed using R version 3.6.0 [15].

## Results

**Fig. 1:**
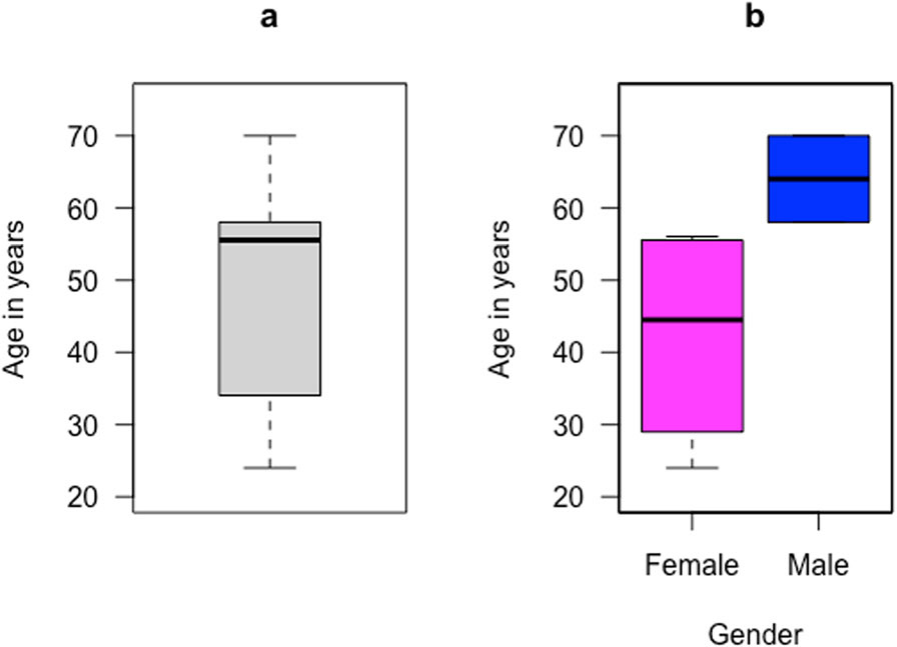
**a** Boxplot that shows the age distribution of HCV positive participants with a median of 55.5 years and interquartile range of 39.25–57.5 years. **b** The age distribution was further stratified by gender and observed in older men (~ 65 years) and in middle-aged women

Study participants were Batswana adults and 63.3% were female. The age ranged from 16 years to 74 years with a median of 44 years and an interquartile range (IQR) of 32–55 years. From the 55 participant samples we had access to, 6 (10.9%) of those were RNA positive as shown in Table 1. The six participants had ages, ranging from 24 to 70 years with a median of 55.5 years as shown in Fig. 1a, and HCV was observed in older males and middle-aged women as shown in Fig. 1b.

**Table 1:** Characteristics of the HCV viraemic positive participants

Sequence ID	Gender	Genotype	Subtype	Accession Number
LB_1	Male	5	5a	MK392625
LB_2	Female	5	5a	MK392626
LB_3	Female	5	5a	MK392627
LB_4	Male	5	5a	MK392628
LB_5	Female	4	4v	MK392629
LB_6	Female	4	4v	MK392630

**Fig. 2:**
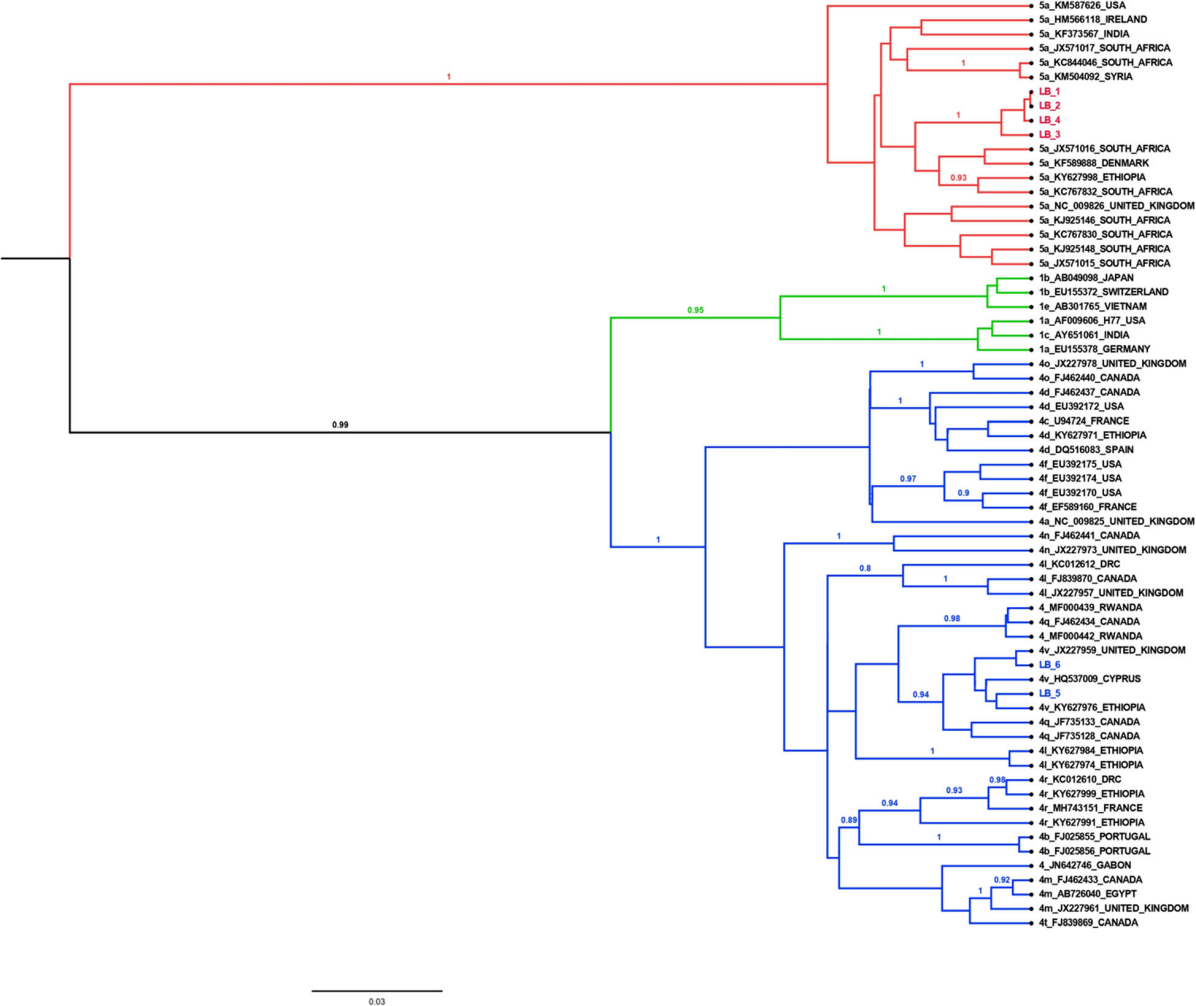
Phylogenetic tree using Bayesian analysis of the 6 amplified Botswana HCV sequences and GenBank references. Reference sequences are labelled with the genotype + accession number + country. The genotype 5a sequences from Botswana are highlighted in red with the prefix LB, while the genotype 4v sequences from Botswana are highlighted in blue with the prefixes LB

Sequence analysis of 6 partial core regions revealed that four of the Botswana sequences belong to genotype 5a, as shown by the clustering with other genotype 5a strains from South Africa, Ethiopia, and Denmark. Two Botswana strains belong to genotype 4v as shown by the clustering pattern with other genotype 4v strains from Ethiopia, Cyprus, and the United Kingdom as shown in Fig. 2.

Further analysis was performed to identify core mutations within the viral sequences as shown in Table 2.

**Table 2:** Core mutations observed in Botswana sequences and their association with disease progression and/or therapeutic response

Mutation	Genotype	Mutation frequency	Clinical relevance	Reference
G95E	4v	1	Not characterized	[16]
R9T	5a	4	Not characterized	[17]
K10Q	5a	4	Increased risk of HCC	[18]
T15I	5a	4	Not characterized	[19, 20]
L36 V	5a	1	Not characterized	[21]
R70Q	5a	4	Increased risk of HCC + poor response to IFN	[17, 22, 23–25]

*Abbreviations*: *HCC* Hepatocellular carcinoma, *IFN* Interferon

Most mutations were observed in genotype 5 samples (R9T, K10Q, T15I, L36 V and R70Q). Only one mutation (G95E) was observed in genotype 4.

## Discussion

This is the first report on the circulating HCV genotypes from Botswana. The finding of genotypes 5a and 4v agrees with previously reported data from southern Africa [5, 26, 27]. Interestingly, genotype 4v has not been observed previously in southern Africa, although other genotypes, such as 4c, 4 g, 4 k, 4q and 4r, have been reported [28, 29]. Genotype 4v has been reported in central Africa and the Middle East. In two studies, genotype 4v was observed in Rwanda and Ethiopia [30, 31]. To achieve elimination of HCV by 2030, DAAs should be introduced and administered to patients as per genotype [10]. According to the observed genotypes, Botswana will need to procure Ledipasvir, Daclatasvir and Sofosbuvir as the DAA regimens recommended to treat genotypes 4 and 5 for patients with and without liver cirrhosis [10]. In this study, the HCV diversity in Botswana is low as compared to South Africa, since only two subtypes were observed. This observation could be due to small sample size and/or varying transmission dynamics.

In the current study the core gene was amplified as the region of interest, despite the recommended NS5B region for HCV genotype classification [32]. We selected a significant fragment of the core region to classify the HCV isolates, which also includes (but is not limited to) putative HCC core related mutations, since the core region has higher amplification rates compared to the NS5B region as previously reported [12].

The core mutations in genotype 5a – R9T, K10Q, T15I, L36 Vand R70Q – account for most of the observed mutations. The K10Q mutation is associated with increased risk of HCC [18]. This mutation was the nucleotide substitution A28C in the core gene bringing about an amino acid substitution from lysine to glutamine. Further analysis from 100 genotype 5 sequences downloaded from the GenBank showed that only 5% of these references contained the K10Q mutation. Interestingly, all four genotype 5 samples from the Botswana sequences had that mutation.

Several studies in genotype 1 strains have indicated that the R70Q mutation increases the risk of HCC due to its oncogenic effect [18, 23–25] and also confers resistance to IFN-based treatments [22]. This mutation occurs due to nucleotide substitution G209A resulting in an amino acid change from arginine to glutamine [18]. From the analysis with the GenBank genotype 5 sequences, it was interesting to note that 81% of the references contained the R70Q mutation compared to 16% with the wildtype amino acid. In a study conducted in South Africa, the R70Q mutation was observed in 90% of genotype 5a blood donors [33]. Whether the impact of this mutation in genotype 1 strains is the same in genotype 5 remains unclear. Presence of this mutation in only genotype 5 isolates could either be due to transmission of a mutated strain or a naturally occurring drug resistant mutation to the SOC [33]. However, the latter assumption cannot be confirmed since there are no data on duration of treatment. Despite the scarce data on impact of this mutation in genotype 5, all four genotype 5 individuals may have a poor response to the current SOC. Thus, DAA regimens may improve treatment of individuals infected with HCV strains harbouring these mutations.

Only one mutation – G95E – was observed in one genotype 4v sample. However, this mutation has not been well characterized or associated with altering viral fitness or drug sensitivity. Most reported mutation analyses have been conducted on genotype 1 strains [20]. Therefore, more longitudinal studies in mutation analysis based on other genotypes, such as 4 and 5, are warranted, as mutations differ by genotype.

A major limitation of the study was the modest population size evaluated and the low amplification success rate; therefore, there was a limited number of positive samples available for analysis. Furthermore, as no HCV antigen enzyme-linked immunosorbent assay (ELISA) was conducted due to low sample volumes, the low amplification rate may reflect a low proportion of current HCV infections versus seropositive but resolved infections. The low amplification rate may also be due to multiple freeze-thaw cycles for sample use in other studies prior to the current analysis. The cross-sectional nature of this study made following up on the patients with HCC-risk factors impossible. Therefore, we could not observe the oncogenic effect of the reported mutations in genotype 5 patients.

## Conclusion

In summary, genotypes 5a and 4v are the circulating genotypes in Botswana. The mutations observed in this study confer resistance to the SOC, and, as per WHO recommendations, there is a need to introduce DAAs as the new SOC for Botswana in order to achieve elimination of HCV by 2030. The DAAs for Botswana HCV patients should include Ledipasvir, Daclatasvir and Sofosbuvir. A longitudinal study with a larger representative population is warranted to develop more understanding of the HCV epidemiology in Botswana.

## Data Availability

The datasets used to support the results of this study are available from the corresponding author upon request. Sequences are available in the National Center for Biotechnology Information (NCBI) GenBank under accession numbers MK392625 to MK392630.

## Abbreviations

DAA: Direct acting antivirals
DNA: Deoxyribose nucleic acid
ELISA: Enzyme-linked immunosorbent assay
HBV: Hepatitis B virus
HCC: Hepatocellular carcinoma
HCV: Hepatitis C virus
HIV: Human immune-deficiency virus
IFN-α: Interferon -alpha
IQR: Interquartile range
MCMC: Markov chain Monte Carlo
NCBI: National Center for Biotechnology Information
RNA: Ribonucleic acid
RT-PCR: Reverse transcriptase polymerase chain reaction
SOC: Standard of care
SVR: Sustained virologic response
WHO: World health organisation
